# HER-2/*neu *and CD117 (c-kit) overexpression in patients with pesticide exposure and extensive stage small cell lung carcinoma (ESSCLC)

**DOI:** 10.1186/1477-3163-4-8

**Published:** 2005-06-09

**Authors:** Anil Potti, Apar Kishor Ganti, Sascha A Tuchman, Kaley Sholes, Eric Langness, Vijay Koka, Michael Koch

**Affiliations:** 1 Department of Medicine, Divisions of Hematology and Oncology, Duke University Medical Center, Durham, NC 27715 USA; 2 Department of Medicine, Section of Oncology-Hematology, University of Nebraska Medical Center, Omaha, NE 68198-7680 USA; 3 Department of Medicine, Duke University Medical Center, Durham, NC 27715 USA; 4 Department of Medicine, University of North Dakota School of Medicine, Fargo, ND 58102 USA; 5 Department of Pathology, Meritcare Medical Center, University of North Dakota School of Medicine, Fargo, ND 58122 USA

**Keywords:** Pesticide exposure, HER-2/*neu *overexpression, CD117 overexpression, c-kit overexpression, extensive stage small cell lung cancer, survival, epidemiology.

## Abstract

**Background:**

The rate of detection of HER-2/*neu *and CD117 (c-kit) overexpression in small cell lung cancer (SCLC) has varied widely; between 5–35% and 21–70% respectively.

**Methods:**

To evaluate the relationship between pesticide exposure and HER-2/*neu *and CD117 overexpression in extensive stage SCLC (ESSCLC), we identified patients with ESSCLC and assessed pesticide exposure using a predetermined questionnaire. An exposure index (hours/day × days/year × years) ≥ 2400 hours was considered as 'exposed.' HER-2/*neu *overexpression was evaluated on archival tissue using the DAKO Hercep test, and CD117 testing was performed using immunohistochemistry (A4052 polyclonal antibody).

**Results:**

193 ESSCLC patients were identified. Pesticide exposure data could be obtained on 174 patients (84 females and 109 males) with a mean age of 68.5 years. 53/174 (30.4%) revealed HER-2/*neu *overexpression. 54/174 (31.03%) specimens showed CD117 overexpression by IHC. On multivariate analysis, HER-2/*neu *overexpression was associated with diminished survival (p < 0.001). In comparison, CD117 expression did not have an adverse prognostic value (p = 0.025). 41/53 (77.4%) patients with HER-2/*neu *overexpression and 47/121 (38.8%) patients without overexpression had exposure to pesticides (odds ratio: 5.38; p < 0.01). Among the cohort tested for CD117, 29/54 (53.7%) patients with CD117 overexpression and 59/120 (49.2%) patients without CD117 overexpression had pesticide exposure (odds ratio: 1.18; p = 0.12).

**Conclusion:**

Pesticide exposure affects HER-2/*neu *but not CD117 overexpression. Future studies are needed to determine specific pesticide(s)/pesticide components that are responsible for HER-2/*neu *overexpression in ESSCLC, and to validate our findings in other solid tumors that overexpress HER-2/*neu*.

## Introduction

Lung cancer is the most common malignancy both in terms of incidence and mortality in the world. In 1999, there were 1.04 million new cases (12.8%) and 921,000 deaths (17.8%) due to lung cancer all over the world [1]. It is expected that in 2004, there will be 173,770 new cases of lung cancer diagnosed and 160,440 people will die of lung cancer in the United States [2]. Small cell lung carcinoma (SCLC), which comprises of 20% of lung cancer, is characterized by a rapid growth rate and early metastases [3,4]. Despite recent advances in therapy, the 2-year survival in limited stage small cell lung cancer is only 20–30% [4], while the median survival in extensive stage small cell lung cancer (ESSCLC) is less than a year (10.1 months) [5]. Given these dismal survival rates, it is imperative that factors like biomarker profiles be evaluated in an attempt to define survival characteristics.

We had previously described the overexpression of HER-2/*neu *in approximately 29% of patients with ESSCLC. In that same study, we also showed that this overexpression led to a poorer outcome [6]. Micke et al found a definite amplification of c-erbB-2 oncogene in 13% of patients with SCLC; predominantly in ESSCLC. They thereby suggested that HER-2/*neu *overexpression was more important in advanced stages of the disease [7]. In contrast, however, Schneider and associates found minimal or no overexpression of HER-2/*neu *in four out of four cell lines derived from small cell lung cancer [8]. Similarly, Bunn et al evaluated eleven small cell lung cancer cell lines and found no evidence of HER-2/*neu *overexpression [9].

In trying to explain these differences, we had hypothesized in our previous report that the difference in the methods used to detect HER-2/neu status (immunohistochemistry vs. fluorescent in situ hybridization) and the population studied (exclusively ESSCLC in our study), could have been responsible for the difference found in overexpression [6]. However, we were unable to definitively explain the possible mechanisms responsible for this high rate of HER-2/*neu *overexpression seen in our study.

We have also demonstrated earlier that CD117 (c-kit) overexpression occurred in about a third of patients with ESSCLC [10]. The proto-oncogene c-kit encodes a transmembrane tyrosine kinase receptor (c-KIT/CD117) related to the platelet-derived growth factor (PDGF)/colony-stimulating factor-1 (CSF-1) (c-fms) receptor subfamily [11]. CD117 is thought to play an important role in hematopoiesis, spermatogenesis, melanogenesis and, more recently, in carcinogenesis [12-14]. Overexpression of CD117 has previously been documented in myeloid leukemia, neuroblastoma, breast tumor, colon tumors, gynecological tumors, testicular germ cell tumors and SCLC [15-18].

Presented with two potential targets for therapy and/or possible predictors of survival, the objective for our current work was to undertake a retrospective comparison of the degree of overexpression of HER-2/*neu *and CD117 in ESSCLC samples, with a possible link to prior pesticide exposure in our largely rural study population.

## Materials and methods

After approval from the Institutional Review Board and the Human Subjects Committee, a two-phase retrospective study was performed. The records of all patients with a diagnosis of SCLC from January 1991 through April 2001 were reviewed. The data collected included the following variables regarding the patient's cancer history: age, sex, socioeconomic status, family history of cancer (if positive, type of cancer), smoking, performance score (ECOG), stage of cancer, treatment modality/modalities for SCLC, metastatic sites (other organs) involved, recurrent disease: symptoms at first recurrence age of recurrence, months from initial diagnosis and stage of disease at recurrence, treatment received for the recurrence, cause of death, other co-morbidities (non-malignant illnesses) and associated malignancies (if any) in the same patient. In addition, we conducted a population-based epidemiologic study in an effort to establish a relationship, if any, between pesticide exposure, ESSCLC and HER-2/*neu *or CD117 overexpression.

Pesticide risk assessment interviews were performed (by a single member of the team for consistency) via telephone on the basis of a pre-determined questionnaire. The questionnaire investigated occupations and hobbies with special emphasis on:

(a) nature of exposure: insecticides and herbicides, organic solvents (paints, varnishes, solvents and glues) and petroleum products (diesels, petrol, oils, greases, dyes, inks and colorings),

(b) type of exposure (preparation and/or spraying of pesticide solutions, direct handling of solvents containing materials or petroleum products),

(c) use of protective measures in the workplace (dissolving or spraying the pesticide with pressurized containers, using glues or varnishes with adequate ventilation, etc.). The subject was considered as 'non-exposed,' in the presence of these effective protection measures.

(d) duration of exposure. An exposure index was calculated for each interviewed subject according to the following formula: hours/day × days/year × years [19]. Patients with an exposure index >2400 h were considered as 'exposed', since previous reports have indicated that this figure represents heavy exposure to genotoxic agents [20,21].

HER-2/*neu *oncoprotein overexpression was assessed by immunohistochemistry (IHC). Adequate pathologic samples were available on 193 subjects. IHC staining was carried out on formalin fixed, paraffin-embedded material, using the Hercep test developed by DAKO^® ^Immunostaining was classified as follows: 0 = no staining; 1+ = faint, incomplete membranous pattern; 2+ = moderate, complete membranous pattern; 3+ = strong membranous pattern [22]. A trained pathologist (M.K.), who was blinded from the clinical and exposure history of the patient, interpreted the IHC results. An IHC score of 2+ or greater was considered as positive for HER-2/*neu *overexpression [23,24].

Detection of CD117 overexpression was assayed using an IHC technique on archival paraffin-embedded tissue specimens. Results were recorded on a semi-quantitative scale to record the number of positive cells (10%, 10–50% and >50%) and the intensity of reaction. CD117 status was reported as positive if it was >10% and negative if it was <10%. Immunohistochemical staining for c-kit (CD117) was performed using a 1:250 dilution of the rabbit polyclonal antibody A4502 (IMPATH, Los Angeles, CA, USA) with the EnVision detection system (IMPATH). An antigen retrieval method was not utilized. Appropriate positive and negative controls were used throughout the testing process. We used the A4502 antibody because it has shown consistent performance with a low background, because it seems to be the most widely used c-kit antibody, and because it is the antibody specified for CD117 testing in the large cooperative clinical trials of selective tyrosine kinase inhibitor STI-571. All interpretation of CD117 testing was performed by a single pathologist (M.K.) who was blinded from the clinical data of patients in our study population.

Statistical analysis was carried out using the chi-square test to evaluate the association between pesticide exposure and HER-2/*neu *vs. CD117 overexpression, and the Mann Whitney U test to assess the impact of HER-2/*neu *and CD117 overexpression on survival. Overall survival was calculated from the date of diagnosis of lung carcinoma by the Kaplan-Meier product limit method [25]. Logistic regression analysis was performed to estimate the magnitude of the effect of pesticide exposure on HER-2/*neu *and CD117 status. A multivariate analysis was performed to analyze the prognostic impact of overexpression of either oncoproteins. Statistical analysis was performed using SPSS-10^®^

## Results

Between 1991 and 2001, 223 patients with ESSCLC were identified of whom 209 patients had complete records of treatment. The most common chemotherapeutic regimen involved using either cisplatin/carboplatin and etoposide or carboplatin and paclitaxel. The most common second-line agent used was docetaxel. 174 patients (98 males, 76 females) with a mean age of 68.5 years (range: 42–90 years) had adequate pathological specimens available for definitive HER-2/*neu *and CD117 testing by IHC. The major symptoms at presentation and other relevant demographic data of the study population are described in Table-1. In our study, weight loss (52.8%) and cough (59.0%) were the two most common presenting complaints that showed a trend towards a decreased survival (p = 0.03) in patients with ESSCLC.

**Table 1:** Demographic data and symptoms at initial presentation in relation to HER-2/*neu *and CD117 overexpression (n = 174)*.

	**HER-2/*neu *negative (n = 120)**	**HER-2/*neu *positive (n = 53)**	**CD117 negative (n = 119)**	**CD117 positive (n = 54)**
**Mean age at diagnosis (years):**	65	66	63	65.5

**Mean age at death (years):**	67	68	67.5	67

**Sex:**				
• Male (n = 98)	62	36	65	33
• Female (n = 76)	59	17	65	21

**Performance score:**				
• Scores 0 – 2 (n = 102)	71	32	69	33
• Scores 3 – 4 (n = 72)	49	21	50	21

Most common symptoms*:				
• Weight loss (n = 61)	47	14	44	17
• Cough (n = 53)	36	17	33	20
• Dyspnea (n = 33)	26	7	27	6

* not all symptoms are included and some patients had more than one symptom at presentation.

Fifty-three (30.4%) of the 174 specimens revealed HER-2/*neu *overexpression by IHC while the remaining 121 (69.6%) did not (Table 1). After adjusting for age, smoking, ECOG score and treatment, HER-2/*neu *overexpression was associated with a statistically significant diminished survival (p < 0.001) [6]. Of the 53 patients with HER-2/*neu *overexpression, we were able to ascertain pesticide exposure in 41 patients (77.4%), while of the 121 without HER-2/*neu *overexpression, 47 (38.8%) had a history of significant pesticide exposure, a statistically significant difference (odds ratio: 5.38; p < 0.01, 95% CI: 2.5 – 11.2). After adjusting for age, gender and smoking history, the odds ratio for developing a HER-2/neu positive tumor following pesticide exposure was still significant (odds ratio: 15.7; 95% CI 5.3 – 46.6; p < 0.01). The pesticide exposure occurred any time between 2 and 21 years prior to the diagnosis of the lung cancer. Patients with an exposure index >2400 h were considered as exposed although the length of exposure varied from 2400 to 7100 hrs.

**Figure 1:**
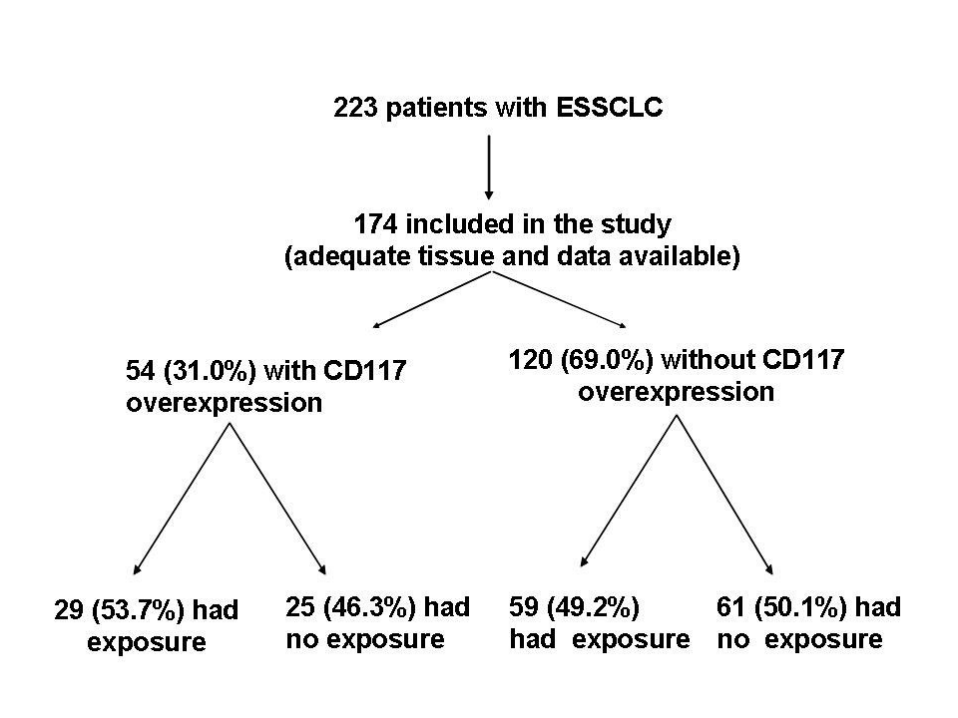
Characteristics of CD117 overexpression and pesticide exposure in extensive stage small cell lung carcinoma (ESSCLC).

As for CD117, of the 174 available specimens for review, 54 of 174 (31.0%) demonstrated IHC-verifiable CD117 overexpression, whereas 120 (69.0%) did not. Twenty-nine of the 54 patients overexpressing CD117 (53.7%) had evidence of pesticide exposure, whereas 59 of the 120 patients devoid of CD117 overexpression (49.2%) also had pesticide exposure (odds ratio 1.18; p = 0.12) (Figure 1). Moreover, in contrast to HER-2/*neu*, CD117 overexpression carried with it no adverse prognostic significance (p = 0.025).

## Discussion

HER-2/*neu *(also known as c-*erb*B-2) oncogene is the second member of the epidermal growth factor receptor family. It is overexpressed in many different types of human malignancies, notably, breast, lung, ovarian, gastric, pancreatic, colorectal, and cancers of the female genital tract [26-33]. HER-2/*neu *overexpression seems to induce chemoresistance in certain experimental conditions. Also, HER-2/*neu *overexpression has been associated with a poor overall survival and the development of metastatic phenotypes [26]. These findings suggest that HER-2/*neu *may serve as an excellent target for developing targeted anticancer agents, and indeed, the benefit of the monoclonal antibody against HER-2/*neu *(trastuzumab) has been demonstrated in the treatment of metastatic breast carcinoma [34].

CD117 (c-kit) is a receptor-tyrosine kinase involved in regulation of hematopoiesis as well as cell migration of germ cells. Mutations in several key exons give rise to constitutive activation of the receptor that ultimately is responsible for the carcinogenesis of gastrointestinal stromal tumors. This finding has in part led to the creation of novel, tailored molecular therapy in the form of imatinib mesylate (STI571) that has been successful where many other anti-cancer therapies have failed [35].

Although animal studies demonstrate that many pesticides are carcinogenic, (e.g., organochlorines, creosote, and sulfallate) while others (notably, the organochlorines DDT, chlordane, and lindane) are tumor promoters, human data, however, are limited by the small number of studies that evaluate individual pesticides [36]. In a case control conducted to evaluate the hypothesis that past exposure to the pesticide lead arsenate led to an excess mortality from respiratory cancer, Wicklund et al found that the presence, the intensity, nor the duration of exposure to lead arsenate differed between cases and control subjects [37]. McDuffie et al reported an absence of correlation of lung cancer risk with occupational exposure to any specific pesticide or pesticides grouped by chemical composition [38]. In contrast, Blair et al studied the mortality experience of a cohort of 3,827 white men licensed to apply pesticides in Florida to investigate health effects associated with chronic exposure to pesticides. They found an increasing risk of lung cancer with number of years licensed suggesting that some pesticides may be carcinogenic in humans [39]. Similarly, De Stefani et al found that workers employed in the construction industry, as well as those exposed to DDT may have an excess risk of lung cancer [40]. Becher and associates also showed an increased overall cancer mortality and mortality of respiratory cancer after long-term exposure to phenoxy herbicides and dioxins [41]. Pesatori and co-workers showed that the risk of lung cancer was greater among those who worked as pest control operators than non-pest control workers [42].

In a predominantly agricultural state like North Dakota, where farming is the main occupation, exposure to various pesticides and herbicides is an everyday occurrence. Tessier and Matsumara have shown that in the human prostate cancer cell lines LNCaP and PC-3, erbB-2 kinase was activated by pesticides of different chemical classes: the organochlorine insecticides beta-hexa-chlorocyclohexane (beta-HCH), o,p'-dichlorodiphenyltrichloroethane (o,p'-DDT), heptachlor epoxide, the pyrethroid insecticide trans-permethrin, and the fungicide chlorothalonil [43]. Similarly, in another study, Enan and Matsumara examined the effect of o,p'-DDT, the most estrogenic congener of the DDT family of chemicals and beta-HCH on protein phosphorylation activities in MCF-7, a line derived from human breast cancer cells. They found that o,p'-DDT activated c-neu (HER-2/*neu *product protein) at extremely low concentrations [44]. In a previous report, we had shown that pesticide exposure was associated with HER-2/neu overexpression [45].

With regards to CD117 overexpression, our prior work has demonstrated that CD117 is overexpressed in about 30% of patients with ESSCLC [10]. Although the techniques to ascertain CD117 testing were different, since our reported prevalence of CD117 overexpression was different from that described in previous studies [46,47], and the fact that our study population was predominantly rural and mostly consisted on of the farming community, we decided to explore the link between CD117 overexpression and pesticide exposure. In addition, despite the evidence on the role of pesticides in the pathogenesis of lung cancer via potential avenues such as HER-2/*neu*, the exact molecular role of pesticides on tumorigenesis in humans is unclear at this time.

Although there are no studies in lung cancer, either in patients or on cell lines demonstrating the biologic effects of pesticides or their components, our results indicate that pesticide exposure may indeed be related to HER-2/*neu *but not CD117 overexpression in our select population with ESSCLC. We found that patients with HER-2/*neu *overexpression were significantly more likely to have been exposed to pesticides (41/53; 77.4%), than those without HER-2/*neu *overexpression (47/121; 38.8%) (Odds ratio: 5.38; p < 0.01). Our findings seem to agree with the field cancerization theory of Gazdar which states that all or much of the aerodigestive tract epithelium has been mutagenized, perhaps as the result of exposure to carcinogens leading to multiple molecular changes in individual cells ultimately resulting in carcinogenesis [48]. Although there is a relative dearth of clinical studies, experimental studies using cancer cell lines revealed that HER-2/*neu *was activated by various insecticides and fungicides of different chemical classes [43,44]. However, whether this activation translates into clinical malignancy has not been studied thus far. Our data suggest that the findings of these experimental models may in fact be true in the carcinogenesis of small cell lung cancer. Interestingly, however, CD117 overexpression was not more prevalent in patients with pesticide exposure (29/54; 53.7%) than those lacking such exposure (59/120; 49.2%) (Odds ratio 1.18, p = 0.12).

Thus, the difference in our reported prevalence of CD117 overexpression in ESSCLC and previous studies possibly was not due to pesticide exposure. Hibi et al. [47] reported that over 60% of cases with SCLC overexpress CD117. In another series, Sekido et al. [46] reported a higher incidence of CD117 overexpression (81%) in SCLC. The reason for the difference in our findings and the previous reports may be attributable to the fact that our large study included only patients with ESSCLC. Although the presence of CD117 does not carry definite prognostic value, it is possible that overexpression may be preferentially observed in patients with limited-stage SCLC. Earlier reports used northern or western blotting techniques for assessing CD117 expression; both of which are more sensitive but less specific than IHC techniques [49,50]. This difference in methodologies may also contribute to the observed difference in CD117 overexpression.

We realize that as with most questionnaire-based studies, a drawback of our study-design is the distinct possibility of a recall bias among the case population. In addition, in cases where the patient was deceased, the data was obtained from the next of kin, who may or may not have had detailed knowledge regarding pesticide exposure of the subject in question. Interviewing technique plays a central role in improving accuracy of the recall and is under the control of the investigator [51]. We tried to minimize the effect of interviewing technique on the results by having one single investigator conduct all the interviews. Also, we used prompted questions, rather than open-ended question during the collection of exposure-data as that has been shown to decrease recall bias [52]. Finally, another potential drawback to our study was the fact that although we gathered enough data regarding the extent of pesticide (insecticide/herbicide) exposure, while implementing our questionnaire, we failed to investigate the extent of exposure to specific pesticides/their components. However, it is possible that the effect of individual pesticides/pesticide components on HER-2/*neu *overexpression may be better evaluated initially using SCLC cell lines [44].

In conclusion, HER-2/*neu *overexpression was seen in slightly less than a third of patients with ESSCLC, and was associated with decreased survival. Conversely, CD117 overexpression was noted in approximately half of ESSCLC patients, but was not correlated to prognosis. Our data also suggest that pesticide exposure seems to be related to HER-2/*neu *overexpression and may explain the increased incidence of overexpression detected in our study population. However CD117 expression appears to be uninfluenced by pesticides, at least at the level of exposure analyzed in our data. Future epidemiologic studies are needed to validate our findings, and also to investigate which pesticide(s)/pesticide components are actually related to HER-2/*neu *overexpression, so that proper preventative measures may be adopted. Moreover, despite the lack of an evident relation between pesticides, prognosis, and CD117, this cell marker may nonetheless provide a valuable therapeutic target for select patients with ESSCLC as it does for certain patients with gastrointestinal stromal tumors. Further studies are warranted to investigate that possibility.
